# Real-world experience with insulin glargine U300 in pediatric type 1 diabetes: glycemic control, insulin requirements, and patient-reported outcomes

**DOI:** 10.1186/s12902-026-02279-x

**Published:** 2026-04-17

**Authors:** Ilayda Altun, Didem Güneş Kaya, Mert Uçar, Yakup Gözderesi, Gökçe Velioğlu Haşlak, Hasan Karakaş, Elvan Bayramoğlu, Olcay Evliyaoglu, Hande Turan

**Affiliations:** Department of Pediatric Endocrinology, Cerrahpasa Medical Faculty, Istanbul University Cerrahpasa, Fatih, Istanbul, Türkiye

**Keywords:** Diabetes mellitus, type 1, Insulin glargine U100, Insulin glargine U300, Glycemic control, Pediatric quality of life inventory, Numeric rating scale

## Abstract

**Background:**

Achieving optimal glycemic control in children with type 1 diabetes(T1D) is challenging. Insulin glargine U300 offers a flatter pharmacodynamic profile and longer duration of action than glargine U100, but pediatric real-world data are limited. This study evaluated the effects of switching from U100 to U300 on glycemic control, body composition, pain, and quality of life.

**Methods:**

This prospective, single-center observational study included children aged 6–20 years with T1D who switched from U100 to U300. Assessments were performed at baseline, week12, and week24. The primary endpoint was change in HbA1c; secondary endpoints included insulin dose(U/kg), hypoglycemia frequency, 3:00 a.m. glucose, lipid parameters, body composition, Numeric Rating Scale (NRS) for pain, and Pediatric Quality of Life Inventory (PedsQL) scores. Participants were stratified by baseline HbA1c (< 9% vs. ≥ 9%).

**Results:**

Sixty-three participants (mean age 14.98 ± 3.53 years) were analyzed. Basal insulin dose increased significantly from 0.48 ± 0.12 U/kg/day on Gla-100 to 0.51 ± 0.14 and 0.51 ± 0.15 U/kg/day at weeks 12 and 24, respectively (*p* < 0.001). HbA1c levels demonstrated a small but statistically significant change during follow-up (*p* = 0.048). (8.20 (5.9–14.8) %→ 8.00 (6.0–12.2) %→ 8.25 (5.7–12.6) %). Participants with HbA1c ≥ 9% showed a significant reduction(ΔHbA1c − 0.50 (− 3.80–1.00) %; *p* = 0.014), while those < 9% had a mild increase(ΔHbA1c 0.10 (− 0.90–5.30) %). Night-time (03:00 AM) blood glucose levels decreased significantly across the three time points (*p* = 0.007), more prominently in the higher HbA1c group. NRS improved markedly (*p* < 0.001), though PedsQL scores were unchanged.

**Conclusions:**

Switching from U100 to U300 maintained glycemic control with modest insulin dose increases. Greater benefits were observed in patients with higher baseline HbA1c, alongside reduced nocturnal glucose and injection pain.U300 appears to enhance comfort and nighttime stability in pediatric T1D.

**Clinical trial number:**

Not applicable.

**Supplementary Information:**

The online version contains supplementary material available at 10.1186/s12902-026-02279-x.

## Abstract (significance)

What is known

What is new

- Insulin glargine U300 provides a flatter and more prolonged pharmacokinetic and pharmacodynamic profile compared with U100, leading to improved glycemic stability and a lower risk of hypoglycemia in adults with type 1 diabetes.
- Evidence on the efficacy, safety, and patient-reported outcomes of switching from Gla-100 to Gla-300 in pediatric populations remains limited.

- This prospective real-world pediatric study is the important to evaluate patient-reported measures (injection-site pain and quality of life) following a switch from Gla 100 to Gla 300.
- Transitioning to U 300 maintained overall HbA1c levels while significantly improving glycemic control in patients with poor baseline control (HbA1c ≥ 9%), accompanied by a modest increase in basal insulin requirements.

**Supplementary Information:**

The online version contains supplementary material available at 10.1186/s12902-026-02279-x.

## Background

Type 1 diabetes (T1D) is one of the most common chronic disorders in childhood and adolescence. Its optimal management remains a major challenge. Effective self-management of T1D requires regular blood glucose monitoring, intensive insulin therapy, accurate carbohydrate counting, and appropriate physical activity adjustments. It also depends on the proper management of both hypoglycemia and hyperglycemia, as well as the supportive use of diabetes technologies such as insulin pumps and continuous glucose monitors.

ISPAD [1] recommends a target HbA1c of ≤ 6.5% (48 mmol/mol) when advanced technologies (such as continuous glucose monitoring and insulin pumps) are available. In contrast, the ADA [2] recommends a target HbA1c of < 7% (53 mmol/mol)for most children and adolescents. Both guidelines highlight achieving glycemic targets without causing significant hypoglycemia or impairing quality of life due to fear of hypoglycemia [1, 2]. A target range of 3.9–10 mmol/L (70–180 mg/dL) is recommended, as stable achievement of these targets is critical for reducing the risk of long-term microvascular and macrovascular complications [3].

Despite these recommended targets, most pediatric patients fail to achieve optimal metabolic control in routine clinical practice. Long term glycemic control in T1D remains frequently suboptimal, with HbA1c levels above targets reccurent episodes of both severe hypoglycemia and hyperglycemia. The cross-sectional TEENs study, which included over 8,000 children and adolescents with T1D worldwide, showed that only 27.5% of participants met the HbA1c < 7% target while the overall mean HbA1c was approximately 8.5%. Target achievement decreased with age, from 31.9% in children aged 8–12 years to 29.1% in adolescents aged 13–18 years and only 18.4% in young adults aged 19–25 years [4]. Achieving healthier glycemic outcomes also has significant benefits for healthcare systems and overall economic burden [5]. These findings underscore the persistent gap between guideline recommendations and clinical outcomes, highlighting the need for treatment approaches that can provide durable glycemic stability without increasing the burden of hypoglycemia.

Exogenous ınsulin therapy in T1D is based on the basal-bolus concept (a once-daily injection of long-acting insulin and injections of rapid-acting insulin analogues at mealtime or a continuous subcutaneous insulin infusion(CSII) administered by a programmable pump) and aims to mimics physiologic insulin secretion [6].

The existing literature comparing CSII and multiple daily injections (MDI) demonstrates heterogeneous results. In young individuals with T1D, insulin pump therapy has been associated with a reduced risk of acute metabolic complications compared with insulin injection therapy. Specifically, rates of severe hypoglycemia and hypoglycemic coma were lower among patients treated with pump therapy, with the greatest benefit observed in school-aged children, as reported by Karger et al. [7]. In contrast, a randomized controlled trial by Blair et al. in newly diagnosed pediatric patients found that CSII was neither more clinically effective nor more cost-effective than MDI [8].

Second-generation basal insulins, particularly insulin glargine 300 U/mL (Gla-300), were developed to provide a more stable pharmacokinetic and pharmacodynamic profiles than first-generation basal analogues such as insulin glargine U100 (Gla-100). Compared with Gla-100, Gla-300 produces a smaller subcutaneous depot, a more gradual and prolonged release [9, 10], reduced glycemic variability, and allows greater flexibility in injection timing [4, 11, 12].

The efficacy and safety of Gla-300 in T1D patients were confirmed in EDITION 4 study in adult patients aged ≥ 18 years, Home et al. showed similar glycemic control but a lower risk of severe hypoglycemia compared to Gla-100 In EDITION JUNIOR study in paediatric patients (aged 6–17 years) with T1D, Gla-300 [13, 14] was similar efficacy and safety profiles with a lower risk of severe hypoglycemia and hyperglycaemia with ketosis than Gla-100 [15].

Gla-300 was approved for pediatric use in patients ≥ 6 years aged since November 2019 and became available in Turkey in 2022. However, real-world data in pediatric populations remain scarce. The first observational evidence was provided by Rabbone et al. and the ISPED CARD study, which involved newly diagnosed patients and those previously treated with first-generation basal insulins, respectively [16, 17].

A recent study from Turkey published in 2025 by Tarçın et al. [18] compared continuous glucose monitoring (CGM)–derived sensor metrics during treatment with different insulin glargine formulations, suggesting that Gla-300 may provide more stable basal insulin coverage with potential benefits in reducing hypoglycemia. However, despite these emerging data, evidence remains limited, and further studies are needed to clarify its impact on glycemic stability and insulin requirements in routine pediatric practice.

The present study aimed to evaluate, in a real-world single-center pediatric cohort, changes in glycemic control following the switch from insulin Gla-100 to Gla-300, with the primary outcome defined as the change in HbA1c (%) from baseline to 3 and 6 months after the switch.

Secondary outcomes included changes in total and basal insulin requirements, frequency of hypoglycemia, 03:00 AM and fasting glucose levels, lipid profile parameters, body composition measures, injection-site pain scores, and health-related quality of life scores over the same follow-up period.

## Research design and methods

**Fig. 1:**
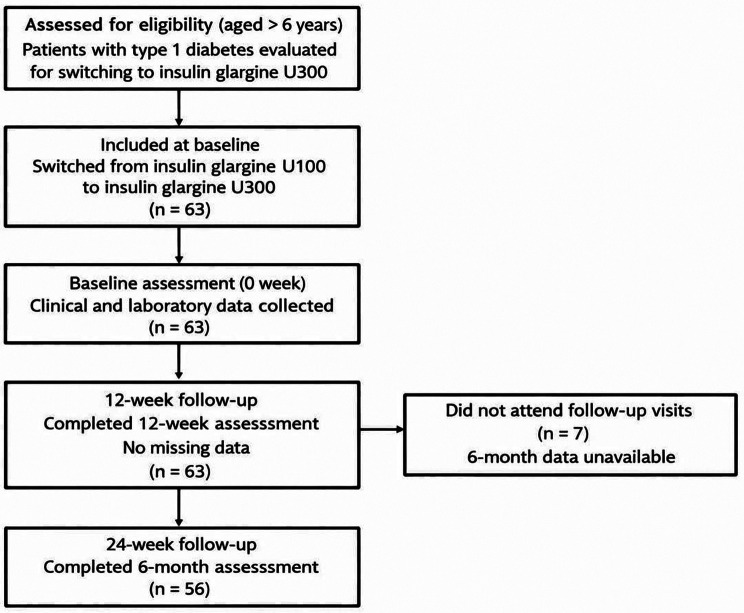
Flow chart of patients inclusion and follow-up

This prospective longitudinal, within-subject observational, single-center study includes children and adolescents aged 6 to 20 years with a diagnosis of T1D who had been treated with Gla-100 and were subsequently switched to Gla-300. All participants were followed prospectively after switching from Gla-100 to Gla-300, and each patient served as their own control. The study population consisted of patients who were followed up at the Department of Pediatric Endocrinology, Istanbul University-Cerrahpasa Medical Faculty, between February 2024 and February 2025. Patients with comorbid metabolic conditions that could affect glucose metabolism—such as chronic kidney disease, chronic liver disease, or cystic fibrosis—were excluded. Exclusion criteria also included patients with type 2 diabetes, those using medications affecting glycemic or lipid metabolism (e.g., metformin, statins), and those with less than 3 months of follow-up. Flowchart showing inclusion and follow-up of children and adolescents with type 1 diabetes switched from insulin glargine Gla-100 to insulin glargine Gla-300 (Fig. 1).

All patients received standardized education [19] in carbohydrate counting, which is a critical component of medical nutrition therapy in diabetes management. This education was delivered by pediatric diabetes dietitian and covered essential topics such as identifying carbohydrate-containing foods, estimating carbohydrate portions, and adjusting insulin doses accordingly. Insulin doses were adjusted according to documented carbohydrate intake records. Regular follow-ups were conducted every 3 months to reassess insulin requirements. The insulin-to-carbohydrate ratio, which determined the appropriate insulin dose for a given amount of carbohydrate intake, was controled and individually adjusted by a clinical dietitian and pediatric endocrinologist based on each patient’s needs.

The data obtained for each patient were age, sex, weight and BMI (body mass index)/SD, and diabetes duration. In each paitent we evaluated glycemic control (Hba1c% (mmol/mol), fasting blood glucose, glucosuria, ketonuria) the number of hypoglycemic events per week, and the average daily insulins doses (basal/total). Additional assessments included lipid profile (total cholesterol, LDL cholesterol, HDL cholesterol, triglycerides) and body composition, measured before switching and at weeks 12 and 24 following the initiation of Gla-300 therapy.

Gla-300 doses were adjusted according to fasting capillary glucose levels and nocturnal (3:00 a.m.) blood glucose levels. All patients were instructed to perform structured self-monitoring of blood glucose, including measurements at preprandial and postprandial time points, as well as at 23:00 and 03:00. Nocturnal blood glucose was assessed by self-monitoring at 03:00 AM on three nights per week, as instructed to all participants and/or their caregivers. None of the participants used CGM during the study period; all patients were followed exclusively using capillary self-monitoring of blood glucose.All patients received either insulin Gla-100 before the switch and Gla-300 after) as a once-daily injection, administered in the evening at the same time throughout the study period. Hypoglycemia was defined according to the ADA 2024 criteria [2] as a documented event per week, identified by a capillary blood glucose level ≤ 70 mg/dL (≤ 3.9 mmol/L) and classified as Level 1 (≤ 70–54 mg/dL), Level 2 (< 54 mg/dL), or Level 3 (severe events requiring assistance).

Body composition parameters—including fat mass, lean body mass, muscle mass, and total body water were measured using bioelectrical impedance analysis device (Tanita MC 780MA; Tokyo/Japan).

The metabolic status of the participants was classified into two groups according to the HbA1c recommendations of the International Society for Pediatric and Adolescent Diabetes: good/moderate control (HbA1c < 9%(75 mmol/mol)) and poor control (HbA1c **≥** 9%) [20].

The PedsQL (Pediatric Quality of Life Inventory) was originally developed by James W. Varni et al. [21] to assess health-related quality of life in children and adolescents aged 2–18 years. The validity and reliability of the Turkish Pediatric Quality of Life Questionniare’in (PedsQL) in 13–18 years old and in 8–12 years –children were evaluated in Turkey by Memik et al. [22, 23]. Scoring was conducted in three domains. With higher total PedsQL scores indicating better perceived health-related quality of life. The Physical Health Summary Score (PHSS / FSTP) was derived from the Physical Functioning subscale with 8 items in the section related to health and activities. The Psychosocial Health Summary Score (PSS / PSTP) was calculated from a total of 15 items, including 5 items each for emotional, social, and school functioning. Total Scale Score (TSS) was obtained by averaging all items in the questionnaire. In the scale prepared according to the five-point Likert system, 0 = never creates a problem, 1 = almost never creates a problem, 2 = sometimes creates a problem, 3 = often creates a problem and 4 = always creates a problem. For total score calculation, a linear conversion was applied and scores were converted to a 0–100 scale. If an item was marked as “never,” it received 100 points; “rarely,” 75 points; “sometimes,” 50 points; “often,” 25 points; and “almost always,” 0 points. Injection-site pain was assessed using the Numeric Rating Scale (NRS), where participants rated their pain on a scale from 0 to 10, with 0 indicating no pain and 10 representing the worst imaginable pain.

PedsQL Scale in Children with Diabetes Mellitus and NRS were introduced to the patients and their parents. Instructions for completion were provided, and adolescents and their families completed the questionnaires online. The first PedsQL questionnair eand injection pain intensity was administered at baseline, before the initiation of Gla-300, and the second assessment was performed 3 months after the switch to Gla-300 therapy.

### Statistical analysis

IBM SPSS Statistics for Mac, version 30.0 (IBM Corp., Armonk, NY, USA) was used for statistical analysis. The Kolmogorov-Smirnov test and Shapiro–Wilk tests was applied to assess the normality of data disturbutions. Descriptive parametric data were expressed as mean, ± standard deviation values and whereas non-parametric data were expressed as median and minimum maximum. Categorical data were expressed as frequency (n) and percentage (%). For comparisons of repeated measurements over time (baseline, 12 and 24 weeks), repeated-measures analysis of variance (RM-ANOVA) was applied for normally distributed variables. When the assumption of sphericity was violated, Greenhouse–Geisser or Huynh–Feldt corrections were used as appropriate. Post-hoc pairwise comparisons were performed using the Bonferroni correction. For non-normally distributed repeated measures, the Friedman test was used, followed by pairwise comparisons when applicable. Paired t-tests were applied for normally distributed paired comparisons, whereas the Wilcoxon signed-rank test was used for non-normally distributed paired data.

Between-group comparisons according to baseline HbA1c levels (< 9% vs. ≥ 9%) were performed using the Mann–Whitney U test, and results were expressed as median (minimum–maximum). A two-sided p value < 0.05 was considered statistically significant.

## Results

### Study populatıon

A total of 63 individuals (*n* = 28 girls / *n* = 35 boys) who had been diagnosed with T1D at a mean age of 8.52 ± 3.92 years met the inclusion criteria and had 6.34 ± 4.3 years T1D duration. The mean age at switch 14.98 ± 3.53 years. Patients who switched to Gla-300 were followed for 12 and 24 weeks, and the data are summarized in Table 1. At 12 weeks, all participants(100%) had evaluable data whereas at 24 weeks data were available for 56 participants (86%) (Fig. 1).

**Table 1:** Baseline demographic and clinical characteristics of the study population

Variable	Baseline (*n* = 63)
Age at diagnosis (years)	8.52 ± 3.92
Age at switch to Gla-300 (years)	14.98 ± 3.53
Sex (male/female),	35/ 28
Diabetes duration (years)	6.34 ± 4.3
BMI z-score	0.21 ± 1.22
Weight (kg)	56.84 ± 17.06
Total insulin dose (U/kg)	1.02 ± 0.37
Basal insulin dose (U/kg)	0.49 ± 0.13
Basal insulin dose (U)	25 (7–60)
HbA1c (%)	8.20 (5.9–14.8)
HbA1c (mmol/mol)	66 (41–138)
03:00 AM blood glucose (mg/dL)	170 (75–350)
Mean fasting blood glucose (mg/dL)	182 (59–478)
**Lipid profile**	
Total cholesterol (mg/dL)	157.20 ± 31.68
LDL cholesterol (mg/dL)	88.77 ± 27.61
HDL cholesterol (mg/dL)	54 (29–115)
Triglycerides (mg/dL)	76 (32–520)

### Glycemic outcomes

Overall 17.5% of participants met the HbA1c < 7% target while the median HbA1c before the switch was 8.20 (5.9–14.80). HbA1c levels demonstrated a small but statistically significant change during follow-up (*p* = 0.048) (Table 2). Among them, 46 individuals (73%) had HbA1c levels below 9%, and 17 individuals (27%) had HbA1c levels greater than 9%. A significantly greater reduction in HbA1c was observed in the HbA1c ≥ 9% group compared with the HbA1c < 9% group (median − 0.50% vs. 0.10%, *p* = 0.014).The comparison of these two groups is summarized in, Table 3.

**Table 2:** Repeated-measures analysis of demographic, metabolic, treatment satisfaction, and anthropometric parameters in patients who completed 6-month follow-up after switching to insulin glargine U300 (*n* = 56)

Parameter	Time1 Before Treatment (*n* = 56)	Time2 3rd Month on U300 (*n* = 56)	Time 3 6th Month on U300 (*n* = 56)	*p* value
**Primary Outcome**				
HbA1c (%) Mmol/mol	8.20 (5.9–14.8) 66 (41–138)	8.00 (6.0–12.2) 64 (42–110)	8.25 (5.7–12.6) 67 (39–114)	0.048^f^
**Secondary Outcomes**				
**Insulin requirements**				
Total insulin dose (U/kg)	1.04 ± 0.39	1.06 ± 0.28	1.12 ± 0.32	0.043^a^
Basal insulin dose (U)	25 (7–60)	27 (8–80)	–	< 0.001^w^
Basal insulin dose (U/kg)	0.48 ± 0.12^a^	0.51 ± 0.14^b^	0.51 ± 0.15^b^	< 0.001^a^
**Glycemic control**				
03:00 AM blood glucose (mg/dL)	170 (75–350)^a^	160 (98–330)^b^	146 (80–300)^b^	0.007^f^
Mean fasting blood glucose (mg/dL)	182 (59–478)	174 (61–425)	183 (80–576)	0.904^f^
**Acute metabolic events**				
Glycosuria (score)	0 (0–4)	0 (0–4)	1 (0–4)	0.480^f^
Ketonuria (score)	0 (0–3)	0 (0–2)	0 (0–3)	0.024^f^
Level 1 hypoglycemia (episodes/week)	1.10 ± 2.03	0.79 ± 2.08	1.15 ± 2.26	0.873^a^
Level 2 hypoglycemia (episodes/week)	2.11 ± 3.96	1.71 ± 3.32	2.02 ± 3.15	0.750^a^
**Lipid profile**				
Total cholesterol (mg/dL)	155.84 ± 31.4	157.61 ± 37.6	163.054 ± 40.05	0.249^a^
LDL cholesterol (mg/dL)	87.99 ± 26.61	93.00 ± 24.12	94.95 ± 22.79	0.033^a^
HDL cholesterol (mg/dL)	54 (29–115)	53 (25–100)	53 (24–105)	0.666^f^
Triglycerides (mg/dL)	76 (32–520)^a^	92 (33–1072)^b^	87 (32–1318)^ab^	0.040^f^
**Treatment satisfaction scores**				
FSTP	90.62 (40–100)	87.50 (43.75–100)	–	0.249^w^
PSTP	77.80 ± 14.01	78.30 ± 12.07	–	0.761^t^
TS	80.79 ± 11.85	80.79 ± 10.06	–	1.000^t^
NRS	4 (0–8)	3 (0–8)	–	0.001^t^
**Anthropometric & body composition**				
BMI z-score	0.23 ± 1.19	0.26 ± 1.20	0.35 ± 1.23	0.284^a^
Weight (kg)	55.85 ± 17.23^a^	57.59 ± 17.07^b^	59.13 ± 16.61^c^	< 0.001^a^
Fat mass (kg)	11.80 (3.6–31.3)^a^	13.80 (4.0–31.3)^ab^	13.10 (5.0–31.3)^b^	0.003^f^
Lean mass (kg)	20.26 ± 7.31^a^	21.28 ± 7.21^b^	21.17 ± 7.07^c^	< 0.001^a^
Body fluid (kg)	30.25 ± 8.88^a^	31.03 ± 8.70^b^	32.00 ± 8.61^c^	< 0.001^a^
Muscle mass (kg)	40.82 ± 11.59^a^	41.65 ± 11.69^b^	42.72 ± 11.44^c^	< 0.001^a^
Trunk fat (kg)	5.80 (2.2–20.5)^a^	6.80 (2.1–20.5)^ab^	5.80 (2.1–20.5)^b^	< 0.001^f^
Trunk fat (%)	20.16 ± 7.23^a^	21.07 ± 7.12^b^	21.17 ± 7.07^ab^	0.038^a^
Total body fat (%)	22.87 ± 7.50	23.30 ± 7.68	23.27 ± 7.65	0.524^a^

Data are presented as mean ± standard deviation or median (minimum–maximum), as appropriate

Repeated-measures analyses were performed using repeated-measures analysis of variance (RM-ANOVA) or the Friedman test, depending on data distribution. The Wilcoxon signed-rank test and paired samples t-test were used for two-time-point comparisons

Superscript letters (a, b, c) denote results of post-hoc pairwise comparisons with Bonferroni correction; values sharing the same letter are not significantly different, whereas values with different letters indicate statistically significant differences between time points

Statistical tests: a = repeated-measures analysis of variance (RM-ANOVA); f = Friedman test; w = Wilcoxon signed-rank test; t = paired samples t-test

Abbreviations: HbA1c, glycated hemoglobin; FSTP, Final Score of Treatment Perception; PSTP, Perceived Score of Treatment Perception; TS, Total Satisfaction; NRS, Numeric Rating Scale

**Table 3:** Comparison of clinical outcomes when participants were stratified by baseline HbA1c levels (< 9% vs. ≥9%)

Parameter (Δ)	HbA1c < 9% Median (Min–Max) (*n* = 46)	HbA1c ≥ 9% Median (Min–Max) (*n* = 17)	*P* value
Basal Total insulin dose (U/kg)	0.96 (0.244-1.60)	1.11(0.57–2.54)	0.701
Basal insulin dose (U/kg)	0.49 (0.23–0.72)	0.51(0.33–0.84)	0.701
Basal insulin dose (U)	26 (8–60)	28(17–62)	0.701
Basal HbA1c (%)	7.80 (5.90–8.90)	9.8 (9.0-14.80)	< 0.001*
Basal 3:00 AM blood glucose (mg/dL)	198 (110–350)	160(75–320)	0.053
Basal fasting blood glucose (mg/dL)	152(59–373)	250(123–478)	0.04*
Basal TS	81.52(32.60–100.0)	81.52(60.86–100.0)	0.818
Basal PSTP	77.50 (28.33–100.0)	75.0(43.33–100.0)	0.735
Basal FSTP	87.50(40.62–100.0)	90.62 (68.75–100.0)	0.818
ΔTotal insulin dose (U/kg)	0.02 (− 0.61–0.83)	0.01 (− 1.22–0.76)	0.818
ΔBasal insulin dose (U/kg)	0.02 (− 0.07–0.23)	0.03 (− 0.23–0.17)	0.519
ΔBasal insulin dose (U)	1.00 (− 1.00–6.00)	2.00 (0.00–23.00)	0.392
ΔHbA1c (%)	0.10 (− 0.90–5.30)	−0.50 (− 3.80–1.00)	0.014^*^
Δ3:00 AM blood glucose (mg/dL)	−12.00 (− 100–145)	−30.00 (− 140–59)	0.290
Δfasting blood glucose (mg/dL)	−0.50 (− 186–160)	−30.00 (− 207–73)	0.104
ΔLevel 1 hypoglycemia (episodes/week)	0 (-8-10)	0 (-6.0-2.0)	0.739
ΔLevel 2 hypoglycemia (episodes/week)	0.0(-10.0-12.0)	0(-16-2.0)	0.159
ΔTS	0.00 (− 22.83–60.87)	0.00 (− 8.70–10.87)	0.441
ΔPSTP	0.00 (− 26.67–68.33)	0.00 (− 16.67–16.67)	0.996
ΔFSTP	0.00 (− 21.88–46.88)	0.00 (− 9.38–6.25)	0.441
ΔNRS	0.00 (− 4.00–5.00)	−1.00 (− 3.00–1.00)	0.996

Data are presented as median (minimum–maximum). Δ indicates the change from baseline to the 3rd month. Participants were stratified according to baseline HbA1c levels (< 9% vs. ≥9%). Between-group comparisons were performed using the Mann–Whitney U test. P* values < 0.05 were considered statistically significant

Abbreviations: HbA1c, glycated hemoglobin; TS, Total Satisfaction; PSTP, Perceived Score of Treatment Perception; FSTP, Final Score of Treatment Perception; NRS, Numeric Rating Scale

### Insulın dose

Average basal insulin doses and total insulin doses were 0.49 ± 0.13 U/kg and 1.02 ± 0.37U/kg respectively.

Basal insulin dose increased significantly from 0.48 ± 0.12 U/kg/day on Gla-100 to 0.51 ± 0.14 and 0.51 ± 0.15 U/kg/day at weeks 12 and 24, respectively (*p* < 0.001). Also, the total daily insulin dose showed significant change from **1.04** ± 0.39 U/kg/day to **1.06** ± 0.28 and **1.12** ± 0.32U/kg/day at weeks 12 and 24, respectively (*p* = 0.043) (Table 2).

### Nocturnal glucose and hypoglycemia

Night-time (03:00 AM) blood glucose levels decreased significantly across the three time points (*p* = 0.007). No significant changes were observed in mean fasting blood glucose across the study period (*p* = 0.904).

Although reductions in nocturnal (03:00 AM) and fasting blood glucose levels tended to be greater in patients with baseline HbA1c ≥ 9%, these differences did not reach statistical significance (*p* = 0.290 and *p* = 0.104, respectively).

The frequency of level 1 hypoglycemia episodes per week remained stable during follow-up, with no significant differences observed among the three measurements (*p* = 0.154). Similarly, the frequency of level 2 hypoglycemia episodes did not change significantly over time (*p* = 0.751).The frequency of hypoglycemia did not increase during follow-up, with no severe events (level 3) were reported (Table 2).

Notably, numerically larger decreases in nocturnal (03:00 AM) and fasting blood glucose levels were observed in the high HbA1c group, although these differences did not reach statistical significance (median Δ03:00 AM glucose: −30 vs. −12 mg/dL, *p* = 0.290; median Δfasting glucose: −30 vs. −0.5 mg/dL, *p* = 0.104).

### Lipid profile and body composition

With respect to lipid parameters, total cholesterol and HDL cholesterol levels remained unchanged. LDL cholesterol increased modestly over time (*p* = 0.033), and triglyceride levels showed a significant change, with post-hoc analysis indicating a difference between baseline and the 3rd month (*p* = 0.040).

During the 24-week follow-up, body weight increased from 55.85 ± 17.23 kg at baseline to 57.59 ± 17.07 kg at week 12 and 59.13 ± 16.61 kg at week 24, whereas BMI z-scores remained unchanged(*p* = 0.284).

Body composition analysis demonstrated significant increases in lean mass, muscle mass, and body fluid during follow-up. Mean lean mass increased from 20.26 ± 7.31 kg at baseline to 21.28 ± 7.21 kg at the 12th week and 21.17 ± 7.07 kg at the 24th week (*p* < 0.001). Similarly, muscle mass increased from 40.82 ± 11.59 kg to 41.65 ± 11.69 kg and 42.72 ± 11.44 kg, respectively (*p* < 0.001). Total body fluid also showed a significant increase over time, rising from 30.25 ± 8.88 kg at baseline to 31.03 ± 8.70 kg at the 12th week and 32.00 ± 8.61 kg at the 24th week (*p* < 0.001).

Fat mass demonstrated a significant temporal change, with median values increasing from 11.8 kg (3.6–31.3) at baseline to 13.8 kg (4.0–31.3) at the 12th week and 13.1 kg (5.0–31.3) at the 24th week (*p* = 0.003). Trunk fat mass also changed significantly over time (*p* < 0.001), whereas total body fat percentage remained stable throughout follow-up (22.87 ± 7.50%, 23.30 ± 7.68%, and 23.27 ± 7.65%, respectively; *p* = 0.524).

### Patient-reported outcomes

Injection-site pain decreased significantly as measured by the NRS (*p* < 0.001). Health-related quality of life, assessed by the PedsQL (PHSS, PSS, and TSS scores), did not show significant improvement after 12 weeks of follow-up (Table 2).

## Discussion

This prospective, real-world observational study evaluated the metabolic and patient-reported outcomes of switching from Gla-100 to Gla-300 in children and adolescents with type 1 diabetes (T1D).

The results of this study suggest that transitioning to Gla-300 in pediatric patients was associated with several noteworthy outcomes over the 3 and 6 months periods. The main findings were that overall HbA1c decreased significantly following the switch to Gla-300. Patients with poor baseline control (HbA1c ≥ 9%) showed significant improvement. An increase in insulin requirements was observed following the switch to Gla- 300. Total daily insulin dose per kilogram increased significantly over time, accompanied by a significant rise in basal insulin dose both in absolute units and per kilogram. Notably, nocturnal glucose levels decreased both at 12 weeks and 24 weeks. Patient-reported injection-site pain decreased significantly whereas quality-of-life scores remained unchanged.

Meidan HbA1c levels demonstrated a statistically significant change over time. A slight decrease in HbA1c was observed at week 12, followed by a mild increase at week suggesting that initial improvements in glycemic control may not have been fully sustained over time. Our finding of an HbA1c reduction in relation to the value presented from baseline, is similar to the results reported by Nakanishi [24] et al. that the HbA1c levels were decreased in people with T1D, but not to a significant extent. In EDITION JUNIOR study showed the The most pronounced reductions in HbA1c during the initial 12 weeks [15]. In the study by Tarçın et al. [18], no significant differences were observed between Gla-300 and Gla-100 in CGM-derived glycemic metrics, including time in range (TIR) and the glucose management indicator (GMI), although HbA1c levels were not directly compared in that study. Furthermore, in our study, stratification according to baseline HbA1c levels was also performed, and patients with higher baseline HbA1c values experienced a greater mean reduction in HbA1c compared with those in the lower HbA1c categories.

Although Gla-300 was administrated in the evening, the significant increase in basal insulin requirements at both the 3rd and 6th months may reflect dose adjustments needed for optimal glycemic control. Most studies recommend a one-to-one dose conversion when switching from insulin Gla-100 to Gla-300 [16], however, some reports indicate that a modest dose increase of approximately 10–18% may be required to achieve target plasma glucose levels [18, 25]. We found a modest(around 2 U), but statistically significant increase in the median basal insulin dose and mean basal insüline dose /kg/day from baseline to the end of 12th weeks. This finding is consistent with another real-life studies in which patients with T1D were switched from Gla-100 to Gla-300; after 24 weeks, an increase in basal insulin was observed, similar to what was seen in our study [26, 27].

A significant reduction in nocturnal (3:00 AM) blood glucose at the third month may indicate improved overnight glycemic stability. The greater reduction in 3:00 AM blood glucose observed in the HbA1c ≥ 9 group indicates a trend toward improved overnight glycemic control in individuals with poorer baseline glycemic status.

Many patients with T1D do not achieve adequate glycemic control. As a result spending a considerable amount of time outside the euglycaemic range of 3.9–10 mmol/L (70–180 mg/dL). Hypoglycaemia is very commonly observed in T1D and represents a major physiological and psychological barrier to achieving optimal glycaemic control [24]. The beneficial effects of Gla-300 on hypoglycaemia, and particularly severe hypoglycemia events was documented in EDITION 4, EDITION JUNIOR studies and some metaanalysis [14, 15, 28]. A non-significant but clinically relevant reduction in hypoglycemia was observed at week 12 but initial reduction was not maintained at the 24th week. An increase in total insulin doses may partly explain the loss of the initial reduction in hypo glycemia. A more pronounced reduction in hypoglycemia frequency was observed particularly in the group with HbA1c ≥ 9%.

Following the switch to Gla-300, an increase in body weight was observed. This change appeared to coincide mainly with increases in lean mass, muscle mass, and total body fluid. Although slight increases in fat mass and trunk fat were noted, overall body fat percentage, BMI, and BMI z-score remained stable, suggesting that the observed weight change was more reflective of lean tissue expansion rather than excess fat accumulation. One meta-analysis found that children and adolescents with type 1 diabetes have higher body fat percentages compared with healthy peers, and that higher daily insulin doses are associated with greater body fat differences, highlighting the potential role of insulin therapy in shaping body composition [29].

While adult studies have reported less weight gain with Gla-300 compared with Gla-100 at 6 months [30], No comparable pediatric studies are available; therefore, extrapolation of these findings to children and adolescents with type 1 diabetes should be made cautiously, particularly given that weight gain is an expected physiological consequence of normal growth and development in children and adolescents. Moreover, in adolescents with type 1 diabetes, pubertal development is associated with physiological insulin resistance, and even lean mass–predominant weight gain may have metabolic implications that cannot be fully assessed within a short follow-up period.

Given the 6-month duration of follow-up and the absence of direct measures of insulin sensitivity, conclusions regarding long-term metabolic impact should be interpreted cautiously. Overall, our findings suggest a potentially favorable shift in body composition under Gla-300 treatment, characterized by preserved or increased muscle mass, although longer-term studies are required to clarify its implications for insulin resistance and cardiometabolic risk.

Patient-reported parameters were comparable between the two groups, with no statistically significant between-group differences observed. Improvements in health-related quality of life often emerge over longer follow-up periods rather than immediately after treatment changes [31].

Importantly, Gla-300 use was associated with a significant reduction in injection-related pain, which may contribute to improved treatment adherence and comfort. Insulin glargine is formulated as an acidic solution that forms microprecipitates after subcutaneous injection, from which insulin is slowly and continuously released [32]. As the redissolution rate is inversely related to insulin glargine concentration, the higher concentration of Gla-300 results in a smaller injection volume and more compact subcutaneous depot formation [33]. This leads to slower, more stable insulin release and reduced local tissue distension, which may explain the observed reduction in injection-site pain with Gla-300 compared with Gla-100. Adolescents with T1D often report overall quality of life comparable to that of their healthy peers despite daily treatment demands [34], which may explain why improvements in injection comfort do not immediately translate into changes in global quality-of-life scores.

The strengths of our study include its prospective design, the comprehensive assesment of metabolic and patient-reported outcomes, and the focus on a pediatric population for which real-word data are scarce. In addition, the simultaneous evaluation of injection-site pain and health-related quality of life provides complementary patient-centered insights within a real-world pediatric setting.

However, several limitations of this study should be acknowledged. First, the study was conducted at a single center with a relatively small sample size, which may limit the generalizability of the findings. Second, the follow-up period was restricted to 24 weeks, and continuous glucose monitoring was not used systematically; therefore, the frequency of hypoglycemia may have been underestimated. In addition, hypoglycemia events were partly based on self-reported data, which are subject to recall bias. Finally, the absence of a parallel control group (such as patients remaining on Gla − 100 or switching to another basal insulin analog) limits direct comparisons with alternative treatment strategies. Nevertheless, the longitudinal within-subject design, in which each participant served as their own control, partially reduces inter-individual variability and baseline confounding. Accordingly, the results should be interpreted with caution, and future randomized or controlled real-world studies with longer follow-up are warranted to confirm these findings.

## Conclusion

Switching from Gla-100 to Gla-300 was associated with stable overall glycemic control over 24 weeks. In patients with poor baseline control, improvements in glycemic parameters were observed; however, these findings should be interpreted with caution given the study design. Reductions in nocturnal glucose levels and improved injection-site tolerability were also noted. Further studies with larger sample sizes and longer follow-up periods are needed to to confirm and extended findings.

## Supplementary Information

Below is the link to the electronic supplementary material.

Supplementary Material 1

## Data Availability

The datasets generated and analyzed during this studyare available from the corresponding author on reasonable request.

## Abbreviations

ADA: American Diabetes Association
BMI: Body mass index
CSII: Continuous Subcutaneous Insulin Infusion
FSTP / PHSS: Physical Health Summary Score
Gla-100: Insulin glargine U100
Gla-300: Insulin glargine U300
ISPAD: International Society for Pediatric and Adolescent Diabetes
MDI: Multiple Daily Injections
NRS: Numeric Rating Scale
PedsQL: Pediatric Quality of Life Inventory
PSS / PSTP: Psychosocial Health Summary Score
T1D: Type 1 diabetes
TSS: Total Scale Score
