# Supplementary material for: Real-world experience with insulin glargine U300 in pediatric type 1 diabetes: glycemic control, insulin requirements, and patient-reported outcomes
Source: BMC Endocr Disord. 2026 Apr 17;26:166. doi: 10.1186/s12902-026-02279-x (PMC13224483; doi:10.1186/s12902-026-02279-x)
Supplement: Supplementary file 1 — Supplementary Material 1 [file 12902_2026_2279_MOESM1_ESM.docx]

**Supplement1.** Changes in Demographic Characteristics, Insulin Requirements, Glycemic Control, Acute Metabolic Events, Lipid Profile, Treatment Satisfaction, and Anthropometric & Body Composition Parameters From Baseline to the 3rd Month After Switching to Insulin Glargine U300 (n = 63)

| **Parameter** | **Before Treatment (Time 1)(n = 63)** | **3rd Month on U300 (Time 2)(n = 63)** | **Delta**  **(Change)** | **95% Confidence Interval for the Difference (Lower–Upper)** | **P values** |
| --- | --- | --- | --- | --- | --- |
| **Insulin requirements** | | | | | |
| Total insulin dose (U/kg) | 1.02 ± 0.37 | 1.04 ± 0.28 | 0.02±0.30 | -0.05-0.09 | 0.608^t^ |
| Basal insulin dose (U) | 25 (7–60) | 27 (8–80) | 1 (-1.0-6.0) |  | <0.001^w^ |
| Basal insulin dose (U/kg) | 0.49 ± 0.13 | 0.52 ± 0.14 | 0.03±0.06 | 0.01-0.04 | <0.001^t^ |
| **Glycemic control** | | | | | |
| 03:00 AM blood glucose (mg/dL) | 170 (75–350) | 160 (98–330) | -17.0 (-3.80-5.30) |  | 0.017^w^ |
| Mean fasting blood glucose (mg/dL) | 182 (59–478) | 174 (61–425) | -5.0 (-2.7.0-160.0) |  | 0.339^w^ |
| HbA1c (%)  Mmol/l | 8.20 (5.9–14.8)  66 (41–138) | 8.00 (6.0–12.2)  64 (42–110) | -0.10 (-3.80-5.30) | -0.35-0.32 | 0.937^w^ |
| **Acute metabolic events** | | | | | |
| Glycosuria (score) | 0 (0–4) | 0 (0–4) | 0 (-3-3) |  | 0.266^w^ |
| Ketonuria (score) | 0 (0–3) | 0 (0–2) | 0 (-3.0-2.0) |  | 0.621^w^ |
| Level 1 hypoglycemia (episodes/week) | 1.10 ± 2.03 | 0.79 ± 2.08 | -0.30 ±2.68 | -0.00-0.39 | 0.391^t^ |
| Level 2 hypoglycemia (episodes/week) | 2.11 ± 3.96 | 1.71 ± 3.32 | -0.39±4.24 | -1.46-0.67 | 0.461^t^ |
| **Lipid profile** | | | | | |
| Total cholesterol (mg/dL) | 157.20 ± 31.68 | 158.90 ± 37.90 | 1.75± 32.92 | -6.5-10.04 | 0.674^t^ |
| LDL cholesterol (mg/dL) | 88.77 ± 27.61 | 93.66 ± 25.62 | 4.88±18.03 | 0.34-9.43 | 0.035^t^ |
| HDL cholesterol (mg/dL) | 54 (29–115) | 53 (25–100) | 0.0 (-24.10-30.0) |  | 0.786^w^ |
| Triglycerides (mg/dL) | 76 (32–520) | 92 (33–1072) | 4.60 (-188.0-552) |  | 0.146^w^ |
| **Treatment satisfaction scores** | | | | | |
| FSTP | 90.62 (40–100) | 87.50 (43.75–100) | 0.0(-21.88—46.88) |  | 0.452^w^ |
| PSTP | 77.80 ± 14.01 | 78.30 ± 12.07 | 0.50 ±13.03 | -2.78-3.78 | 0.761^t^ |
| TS | 80.79 ± 11.85 | 80.79 ± 10.06 | 0.0±11.0 | -2.77-2.77 | 1^t^ |
| VPS | 4 (0–8) | 3 (0–8) | -1 (-4.0-5.0) |  | <0.001^w^ |
| **Anthropometric & body composition** | | | | | |
| BMI z-score | 0.21 ± 1.22 | 0.25 ± 1.22 | 0.04±0.37 | -0.05-0.13 | 0.404^t^ |
| Weight (kg) | 56.84 ± 17.06 | 58.48 ± 16.91 | 1.64 ± 2.5 | 1.0-2.27 | <0.001^t^ |
| Fat mass (kg) | 11.80 (3.6–31.3) | 13.80 (4.0–31.3) | 0.30 (-8.20-91.0) |  | 0.047^w^ |
| Lean mass (kg) | 20.26 ± 7.31 | 21.28 ± 7.21 | 0.75±2.26 | 0.18-1.32 | 0.010^t^ |
| Body fluid (kg) | 30.66 ± 8.77 | 31.42 ± 8.64 | 0.750. ±1.26 | -3.20-1.07 | <0.001^t^ |
| Muscle mass (kg) | 41.42 ± 11.27 | 42.17 ± 11.34 | 0.74±2.0 | 0.23-1.25 | 0.005^t^ |
| Trunk fat (kg) | 5.80 (2.2–20.5) | 6.80 (2.1–20.5) | 0.30 (-3.10-4.80) |  | <0.001^w^ |
| Trunk fat (%) | 20.26 ± 7.31 | 21.28 ± 7.21 | 1.01-2.56 | 0.37-1.66 | 0.003^t^ |
| Total body fat (%) | 22.67 ± 7.71 | 23.24 ± 7.84 | -0.04-2.46 | -0.04-1.19 | 0.070^t^ |
